# Valorization of Rosehip (*Rosa canina* L.) Pomace Using Unconventional Carbohydrate Carriers for Beverage Obtainment

**DOI:** 10.3390/molecules30010141

**Published:** 2025-01-01

**Authors:** Anna Michalska-Ciechanowska, Jessica Brzezowska, Nancy Nicolet, Kamil Haładyn, Wolfram Manuel Brück, Aleksandra Hendrysiak, Wilfried Andlauer

**Affiliations:** 1 Department of Fruit, Vegetable and Plant Nutraceutical Technology, Faculty of Biotechnology and Food Science, Wrocław University of Environmental and Life Sciences, Chełmońskiego 37 Str., 51-630 Wrocław, Poland; jessica.brzezowska@upwr.edu.pl (J.B.); kamil.haladyn@upwr.edu.pl (K.H.); aleksandra.hendrysiak@upwr.edu.pl (A.H.); 2 Institute of Life Sciences, School of Engineering, University of Applied Sciences and Arts Western Switzerland (HES-SO Valais Wallis), Rue de l’Industrie 19, 1950 Sion, Switzerland; nancy.nicolet@hevs.ch (N.N.); wolfram.bruck@hevs.ch (W.M.B.)

**Keywords:** *Rosa canina* L., rosehip, processing, pomace valorization, drying, powdering, instant products, novel beverages, physico-chemical characteristics, techno-functional properties

## Abstract

Rosehip is of notable scientific interest due to its rich content of bioactives and its wide-ranging applications in nutrition, cosmetics and pharmaceuticals. The valorization of rosehip by-products, such as pomace, is highly significant for promoting sustainability. This study investigates the development of rosehip-based powders and beverage prototypes derived from both juice and pomace to evaluate the potential use of pomace in instant beverage design and compare it with juice-based formulations. Three matrices were evaluated: non-pasteurized and pasteurized juice, as well as non-pasteurized pomace preparations. Powders were produced by freeze- and spray drying using maltodextrin, inulin and unconventional carriers, i.e., palatinose and trehalose. The results demonstrated that carrier addition significantly influenced the physical and techno-functional properties of the powders, such as moisture content (below 10%), water activity (below 0.35), solubility (above 85%), and color indexes (yellowness and browning). The water absorption capacity varied with drying techniques, particularly for inulin-enriched samples, while the matrix type affected the ascorbic acid content. Non-pasteurized pomace powders exhibited a higher antioxidant capacity (67.7 mmol Trolox/100 g dry matter) than their juice counterparts (52.2 mmol Trolox/100 g dry matter), highlighting the potential of the pomace matrix for beverage production. Because of their favorable properties, spray-dried samples were also selected for reconstitution into prototype beverages, among which those obtained from pomace showed a higher antioxidant potential. An analysis of particle sizes, which ranged between 34 nm and 7363 nm, revealed potential interactions between the carrier and matrix, reflected in the distinct behavior of carrier-only samples. Both the carrier type and the matrix significantly contributed to the final properties of the beverages, providing valuable insights for the design of functional food products.

## 1. Introduction

Although not widely recognized until recently, the fruits of the rosehip are now increasingly considered to be one of the most abundant natural reservoirs of multi-target bioactives, with a broad spectrum of health-beneficial effects [1]. Indeed, this fruit is of particular interest to the food, chemical and pharmaceutical industries. In the food sector, rosehip is primarily processed into juices, syrup or wine, among other foods [1]. Furthermore, nutrient-dense extracts derived from this fruit are widely used in various food products, adding new functionality to everyday consumables [2]. For example, rosehip extract has been proposed as a component of an active coating to prolong the shelf life of meat burgers [3]. However, in the majority of studies, the proposed form of rosehip application is a pure extract obtained from high-quality fruit or its juice, while the residual material, including pomace (consisting of seeds, pulp and skins), remains underutilized. Therefore, as with the processing of any fruit, in the case of rosehip it is impossible to eliminate the generation of by-products, and thus many different strategies need to be developed to manage them.

Fortunately, rosehip by-products possess considerable potential for recovery and upscaling; however, the pertinent literature provides only few examples of how to explore these residues for valorization. In the food industry, for instance, rosehip pomace has been added as a functional additive to bakery products, such as shortbread cookies, to enhance their antioxidant activity and simultaneously improve their sensory profile [4]. Additionally, rosehip herbal dust, an industrial by-product from the production of filtered tea, was applied to bread fortification [5]. Also, rosehip puree by-products were used for the production of corn extrudates to improve their nutritional values [6]. Rosehip oil, derived from the seeds in processed pomace, has been used as an ingredient in edible coatings. It effectively suppresses the respiration rate and ethylene production of plums, leading to a two-fold increase in the fruit’s shelf life [7]. In turn, *Rosa roxburghii* L. has been proposed as a substrate for the cultivation of edible fungi, stimulating their growth and leading to a significant increase in protein, amino acid and polysaccharide content [8], thus opening new avenues for the utilization of various rosehip cultivars, including rosehip.

Another flourishing sector of the food industry is the production of functional beverages, including both ready-to-drink and instant products. In line with trends in other sectors, the use of agro-food waste as ingredients is emerging, naturally enriching products and imparting new qualities [9]. To date, different plant by-products, such as sea buckthorn waste, grape and broccoli by-products, have been used for their antioxidant, antimicrobial, anti-inflammatory and nutritional functionalities in soft drinks, infusions and juices [10,11,12]. Nevertheless, the solubility of such components plays an important role, especially for clarified juices and transparent beverages. As such, the use of water-soluble and nutrient-dense extracts derived from various plant by-products appears to be an interesting alternative. For this reason, numerous efforts have been made to optimize the extraction procedures and maximize the recovery yield of bioactives and nutrients, while minimizing adverse environmental and health risks [13]. In a study on the extraction of bioactives from rosehip pomace, the time, the temperature and the concentration of different solvents, including acetone, methanol and water, have been tested [14]. Nevertheless, exploring other food-safe alternatives remains crucial due to significant concerns regarding the suitability of non-GRAS solvents, particularly in terms of food safety and environmental impact. Furthermore, the use of such extracts for beverage production has not yet been thoroughly investigated.

Finally, the form in which the valorized by-products of rosehip are utilized is pivotal for the development of novel foods, whether through their direct incorporation into products or as a basis for instant formulations. Although freeze-drying has long been regarded as the most advantageous technique for preserving the quality of dried products, particularly for thermolabile plant matrices, emerging research highlights the comparable, and in some cases superior, potential of spray drying [15]. Moreover, spray drying is emerging as a preferred method for transforming extracts from food waste into high-quality products. It offers a combination of efficiency, scalability and cost-effectiveness, while preserving bioactive compounds and enhancing functional properties. These advantages position spray drying as a strong competitor to traditional drying techniques in sustainable food production [16]. So far, rosehip matrix has been used for the preparation of teas with the application of freeze- and spray drying [17]. However, there remains a significant lack of studies addressing the microencapsulation of the liquid extracts obtained from its pomace fraction.

It was hypothesized that powdered pomace derived from rosehip processing could compete with rosehip juice, produced under similar conditions, in terms of physico-chemical properties. This makes it a promising candidate for the co-creation of instant functional beverages. Therefore, the aim of this study was two-fold: first, to explore the feasibility of producing powders from pomace and juice preparations through spray drying using unconventional carriers; and second, to evaluate their potential for reconstitution as prototypes of functional beverages.

## 2. Results

### 2.1. Chemical Properties of Preparations

Considering the preparation of products derived from the rosehip juice matrix, pasteurization led to a 2.2-fold decrease in the ascorbic acid content. When considering the matrix type, the non-pasteurized pomace preparation contained a significantly higher ascorbic acid content, comparable to that of the non-pasteurized juice preparation, confirming its similar level to the juice (Table 1). Previously, among various solvents used for the L-ascorbic acid extraction, the highest yield was observed when water was applied to rosehip pomace fraction [14].

The values of the total phenolic compounds were decreasing in the following order: pasteurized juice preparation (P-JP) > non-pasteurized juice preparation (NP-JP) > non-pasteurized pomace preparation (NP-PP). This suggests that components contributing to total phenolic content (TPC) are mainly present in the liquid fraction (juice) rather than pomace, although the pomace still displays comparable levels to juice.

### 2.2. Powder Characteristics

#### 2.2.1. Physical Properties of Powders

Powders gained from rosehip extracts were successfully produced using three types of sugar-free preparations based on juice (pasteurized (P-JP) and non-pasteurized (NP-JP)) and a non-pasteurized pomace preparation (NP-PP) with maltodextrin, trehalose, inulin and palatinose as carriers. In the case of spray drying, based on a previous study [18], the processing parameters were established due to the carriers’ properties, these being mainly palatinose, which allowed for powder production when applying the inlet air temperature of 130 °C. A further increase in inlet air temperature caused stickiness of the products in the drying chamber, making the process impossible to perform and making it impossible to produce fine powders.

The powders obtained had a moisture content (*Mc*) of less than 10% (Table 2), a level recognized as sufficient to ensure microbiological stability in powder formulations [19]. On average, the highest *Mc* was noticed for controls (no carrier addition), regardless of the type of rosehip matrix used for drying. Carrier presence in the liquid feed led to a decrease in *Mc* due to the increase in total solids content [20]. Interestingly, the drying technique affected moisture content differently depending on the carrier type applied. For trehalose and palatinose applications, freeze-drying resulted in products with a higher moisture content, on average almost 30%, compared to spray-dried products (Table 2) [21,22]. When considering matrix type, powders derived from non-pasteurized pomace preparation demonstrated a slightly higher moisture content compared to those obtained from non-pasteurized juice, potentially due to the variations in the soluble and insoluble components between juice- and pomace-based matrices, which may affect the drying process and subsequently influence the *Mc* of the powders. The results indicated that there was no significant correlation between *Mc* of carriers and *Mc* of obtained powders. As previously observed by Michalska-Ciechanowska et al. [23], the lack of a clear relationship between the moisture content of the carriers and that of the powders produced with their addition suggests that the initial moisture level of the carriers has a minimal impact on the final moisture stability of the powders. This trend is similarly evident in the current study.

The water activity (*a_w_*) values of all analyzed samples were below 0.35 (Table 2), indicating that these products may be considered as biochemically and microbiologically stable [24]. In the control samples, pasteurization of the juice resulted in lower *a_w_* values of the powders, an effect observed only in spray-dried products, regardless of the carrier used (Table 2). Similarly to *Mc*, controls had a higher water activity than powders produced with carriers, except for pasteurized juice preparation with palatinose. When the drying techniques were compared, no significant differences were observed between the *a_w_* of powders obtained after freeze- and spray drying. Contrary to previous studies on fruit powders [23], no statistically significant correlation between *Mc* and *a_w_* was found (*r* = 0.524).

The addition of carriers resulted in higher L* coordinate values when compared to the control [25]. Modification of the juice matrix (pasteurization, matrix type: juice/pomace preparation) did not significantly affect the L* values of the powders, nor did the drying technique used for powder production (Table 2). Also, a significant difference in a* and b* values was observed after carrier addition in comparison to non-carrier-added samples (controls). Freeze-drying produced powders with significantly higher a* values than spray drying [18]— over 74% higher for both pasteurized and non-pasteurized juice powders, and 33% higher for pomace preparation powders, regardless of carrier type used. Also, coordinate b* values were significantly higher after freeze-drying, on average 39% higher when compared to spray-dried samples, not considering the carrier type and juice matrix submitted to drying. Moreover, exclusively for spray-dried samples, the coordinate b* values were the lowest for the non-pasteurized juice preparation among the matrices used, not considering the carrier type. Thus, the drying technique had a stronger impact on the L*, a* and b* coordinates than the carrier type used for drying.

The yellowness index (YI, degree of yellowness) and browning index (BI, brown color purity), which can be considered as indicators of non-enzymatic browning in food products [26], were determined for rosehip powders to identify any matrix-related or processing-induced changes. Despite slight differences in the values, the same trend was observed for both parameters, confirmed by a strong positive correlation (*r* = 0.995). When considering only the drying technique (independent of matrix and carrier type), the values for freeze-dried powders were reported to be almost 1.4 times higher for YI and 1.6 times higher for BI compared to spray-dried powders (controls not considered) (Table 2). This may be due to the different structure of the powders, which, in the case of freeze-drying, have a much higher hygroscopicity and therefore a higher moisture content, directly affecting the color, especially the L* parameter (*r* = −1) [27]. Another possible reason could be due to the transformations that take place during the reduction in moisture content, which leads to the condensation of soluble fractions, along with related shifts in pH and oxidation-reduction potential. These changes, due to the relatively longer duration of freeze-drying as opposed to the rapid spray drying technique, are much more likely to promote undesirable non-enzymatic reactions such as the Maillard reaction, caramelization, oxidative degradation or polymerization [28].

On the other hand, considering only the matrix type (independent of drying technique and carrier type), with the exception of palatinose-based powders, the average YI and BI values increased in the following order: NP-JP (38.8 for YI; 36.7 for BI) < NP-PP (45.7 for YI; 44.1 for BI) < P-JP (49.2 for YI; 49.4 for BI) (controls not included). Juice preparation pasteurized prior to subsequent processing (carrier addition, drying) resulted in powders with approximately 1.3-times higher BI and YI values than the same non-pasteurized matrix. This suggests that the non-enzymatic browning reactions are initiated immediately during the pasteurization process (and continue during drying), leading to the formation of brown pigments that, considering, the raw material composition, can be attributed to the degradation of vitamin C or its interaction with amino acids within the matrix [29]. Moreover, despite the above-mentioned, acid-catalyzed sugar decomposition and Maillard-type reactions are recognized as the primary mechanisms driving non-enzymatic browning in fruit juices [30], which in this case can be further intensified and accelerated by the subsequent powdering process.

Regarding the type of carrier used for the preparation of the powders, it was observed that inulin resulted in freeze-dried and spray-dried products with comparable YI and BI values, whereas in the case of other carriers the interplay with the drying technique significantly differentiated these parameters. This may indicate an influence of the composition of the carbohydrate carrier (structure, complexity), which may undergo transformations according to the drying technique used, thus leading to differences in the quality of the powders, as any moisture migration induced by drying may involve changes in the matrix microstructure and consequently the release and/or breakdown of bioactives [29]. In addition, the affinity of the carrier to interacting with specific matrices may also vary depending on the carrier type [31], thus affecting encapsulation effectiveness and consequently determining subsequent alterations, including non-enzymatic browning.

#### 2.2.2. Techno-Functional Properties of Powders

**Figure 1:**
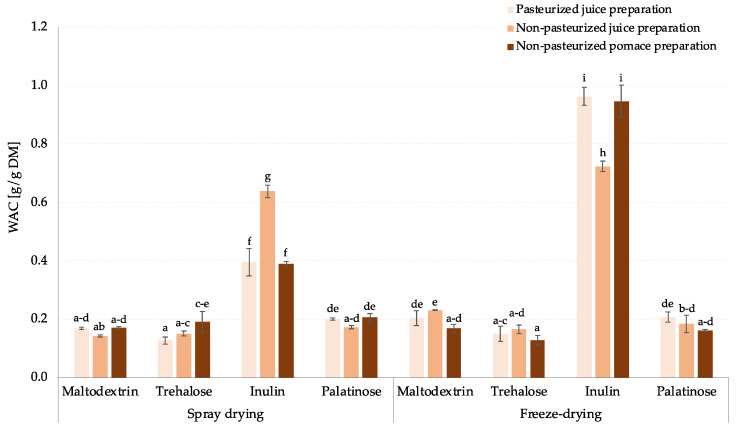
Water absorption capacity [g/g DM] of rosehip powders gained by spray- and freeze-drying with addition of different carrier type; ^a,b,c,…^—different small letters represent statistically significant differences between samples (NIR test; *p* ≤ 0.05).

Although there is limited information in the literature on the water absorption capacity (WAC) of fruit powders, it has been tested in the past. As an example, this parameter was evaluated in pomegranate juice powder fortified with peel phenolic compounds and spray-dried using maltodextrin [32], or powders obtained from pineapple and cashew apple juice, which were dehydrated by foam-mat drying using egg white and soy protein isolate as foaming agents [33], as well as foam-mat freeze-dried date powder with the application of maltodextrin and gum Arabic at 40 and 50% concentration levels [34]. However, there is still a knowledge gap that needs to be filled regarding the comparative analysis of conventional, as well as novel, carbohydrate-based carriers that are widely used in the food industry, especially for the production of spray-dried food powders. As such, the WAC, which reflects the ability of dry foods to absorb water, was analyzed for carrier-added rosehip samples to detect any dissimilarities between powders produced with different carbohydrate carriers (maltodextrin, trehalose, inulin, palatinose) and to verify if the employment of spray- or freeze-drying can additionally influence this techno-functional property. Although the WAC values of the majority of the samples fluctuated around 0.17 g/g dry matter (DM), it was observed that the application of inulin resulted in 2.2 to even 5.6 times higher WAC values, depending on the matrix type and drying technique. As pointed out by Seerangurayar et al. [34], this phenomenon is most likely due to the nature of the carrier, highly evident in this case for inulin, which has been reported to exhibit significantly greater water compatibility than maltodextrin and other disaccharides used [35]. In addition, only for inulin-supplemented powders from pasteurized juice preparation and non-pasteurized pomace preparation, it was reported that freeze-drying yielded products with an approximately 2.4 times higher WAC than spray-dried ones, while powders obtained from non-pasteurized juice preparation scored comparable WAC values (Figure 1). Thus, for the first time, it has been demonstrated that, depending on the matrix used, it is possible to influence the water absorption capacity of inulin-based fruit powders by selecting an appropriate drying technique, while the application of other carriers used in the study ensures a comparable performance in this context.

The water solubility index (WSI) is used to evaluate a powder’s ability to stay uniformly dispersed in water. The food powder should wet rapidly and completely and dissolve smoothly without forming lumps [36].

**Figure 2:**
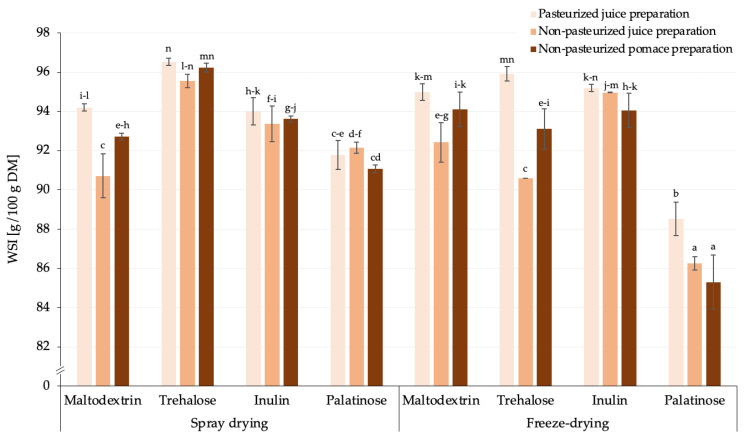
The water solubility index (WSI) of rosehip powders produced with various carriers using spray- and freeze-drying [g/100 g DM]; ^a,b,c,…^—different small letters represent statistically significant differences between samples (NIR test; *p* ≤ 0.05).

Taking into account that the rosehip powders were made by the addition of carriers at the level of 20% (*w*/*w*), the two matrix components, i.e., rosehip preparations (juice/pomace with different chemical composition) and carrier type, may affect the solubility. Previously, among carriers (i.e., inulin, maltodextrin, modified starch, gum Arabic, cellobiose and alginate, added for the spray drying of goldenberry juice), products with inulin had the lowest solubility due to its gel-forming properties [37]. In that study, the lowest WSI was noted for samples produced with palatinose, which was not previously studied in this context. Thus, the various carrier types, with various chemical structures and morphological properties, may have diverse abilities to dissolve in water [38]. Moreover, the interactions that may occur during freeze- and spray drying should also be considered; however, little is known about the effect of these parameters on the solubility of such compositions [39]. Generally, in the case of rosehip powdered products, the pasteurization of the juice before drying resulted in higher values of WSI when maltodextrin and trehalose was applied, regardless of the drying technique used (Figure 2).

Regardless of the carriers used, spray drying resulted in slightly higher average WSI values across samples, irrespective of the type of matrix applied, compared to freeze-drying. A significant difference was observed when palatinose was used as a carrier for freeze-drying. Thus, it can be concluded that the usage of spray drying may moderate the solubility of rosehip preparation powders produced with this carrier, making the products more suitable for reconstitution into soluble products or affecting the accessibility of microencapsulated compounds within a food system [22].

Previously, it was observed that the WSI was not influenced by the spray drying of plant-based samples, nor by maltodextrin concentrations in the liquid feed [40,41]. However, studies indicate that the solubility of plant-based powders with carriers may be modulated by the presence of lipophilic compounds or insoluble components, as well as by the techno-functional properties of the carriers, with certain carriers significantly enhancing WSI values [42]. In cases where the liquid feed contains proteins, the conditions differ, as the drying process itself can induce structural and physicochemical modifications that substantially impact the WSI [43].

In summary, it was demonstrated that spray drying is a viable method for producing rosehip juice and pomace powders made from preparations. The carrier type influenced the techno-functional properties. Spray drying, particularly with palatinose, improved solubility, making it a competitive alternative to freeze-drying for beverage applications. The drying technique notably affected the water absorption capacity, especially with inulin as a carrier.

#### 2.2.3. Chemical Properties of Powders

**Figure 3:**
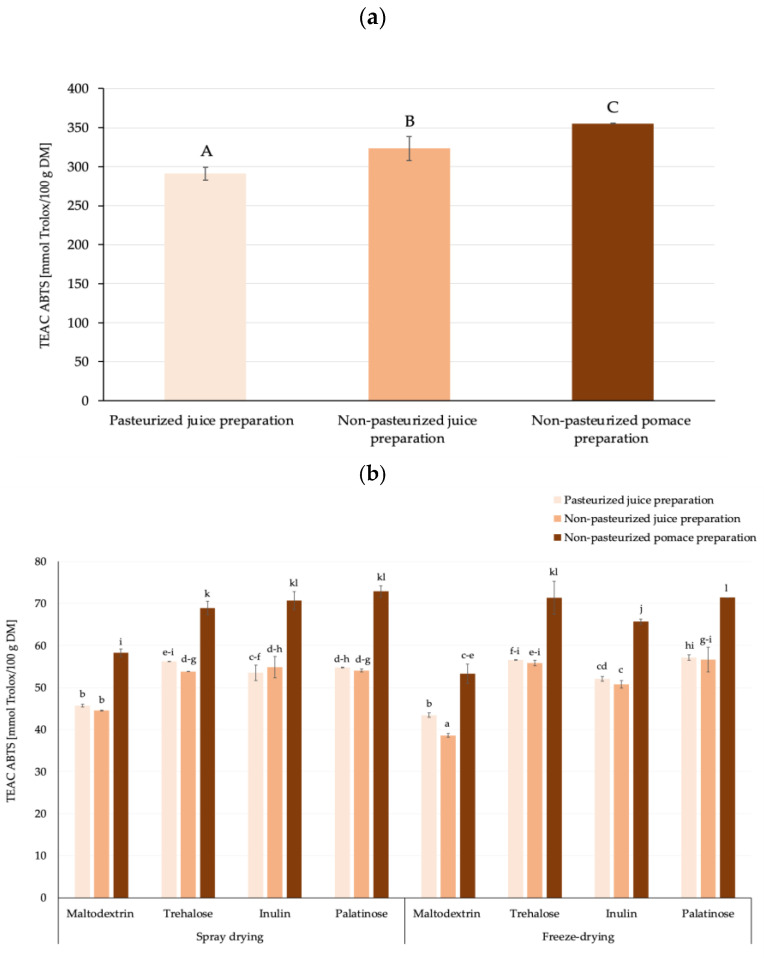
Antioxidant capacity expressed as TEAC ABTS values [mmol Trolox/100 g DM] of rosehip powders (**a**) obtained through freeze-drying without addition of carriers (controls), (**b**) produced by freeze- and spray drying using various carrier agents; ^A,B,C^,^a,b,c,…^—different letters represent statistically significant differences between groups (NIR test; *p* ≤ 0.05).

Among the control samples, the antioxidant capacity values were influenced by both the pasteurization process of the juice and the matrix type used for preparation, leading to the highest ability to scavenge ABTS^•+^ of the pomace-based preparation, which was, on average, almost 16% higher compared to juice preparations. The antioxidant capacity of controls gained from juice (pasteurized and non-pasteurized) and pomace preparations was significantly higher, approx. 5.7-times, when compared to an average TEAC ABTS value gained for juice powders due to the dilution effect of the carriers added (Figure 3a,b). Among the drying techniques used, regardless of the carrier type, spray drying affected the powders’ antioxidant properties to a slightly higher extent than freeze-drying. From the economic standpoint, spray drying is more suitable for powder production, as it was previously stated that freeze-drying is approximately six times more expensive when considering the costs per kg of water removed, when compared to spray drying [44]. Taking both issues into consideration, the powders obtained by spray drying were selected for further study.

**Figure 4:**
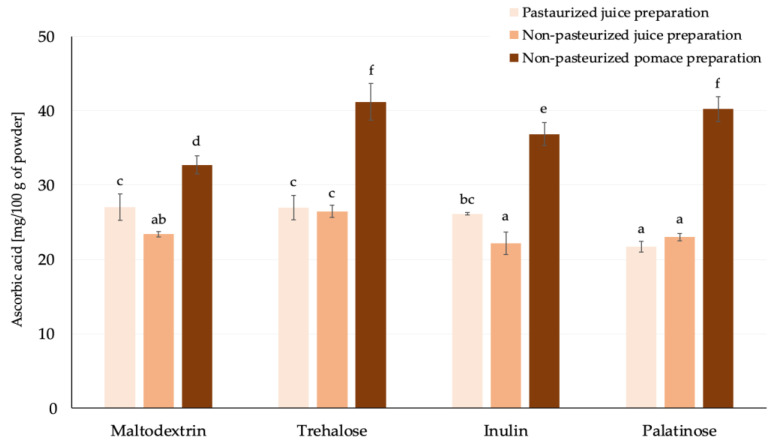
Ascorbic acid content of rosehip powders prepared with various carriers by spray drying [mg/100 g of powder]; ^a,b,c,…^—different small letters represent statistically significant differences between samples (NIR test; *p* ≤ 0.05).

To provide a detailed analysis of the contribution of bioactive compounds to antioxidant capacity, the ascorbic acid content was assessed exclusively in spray-dried products (Figure 4). A strong correlation was observed between TEAC ABTS values and ascorbic acid content (*r* = 0.8524). The matrix type significantly influenced the ascorbic acid content, with powders derived from pomace preparations exhibiting the highest levels among all the analyzed matrix types. On average, regardless of the carrier type used during powder production, the ascorbic acid content in NP-PP was approximately 35% higher compared to powders obtained from juice preparations. Among the carriers applied, the addition of maltodextrin to different types of matrices before drying resulted in the lowest content of ascorbic acid in the powders. A similar observation was made by Igual et al. [45], who used various carrier types in his study. In general, among the carriers used, trehalose resulted in products with the highest ascorbic acid content, indicating that the carrier type affects the stability of ascorbic acid during powder production. This could be attributed to the solubility between ascorbic acid and the carriers, with trehalose demonstrating greater dissolvability compared to maltodextrin, inulin and palatinose.

### 2.3. Characterization of Prototype Beverages Based on Spray-Dried Powders

#### 2.3.1. Antioxidant Capacity in Vitro of Beverages

The formulation of the prototype beverages aimed to develop products that are water-soluble and serve as sources of bioactive compounds with health-promoting properties. This approach sought to evaluate the potential for valorizing pomace-derived preparations by transforming industrial by-products into food ingredients with potential applications as beverages.

**Figure 5:**
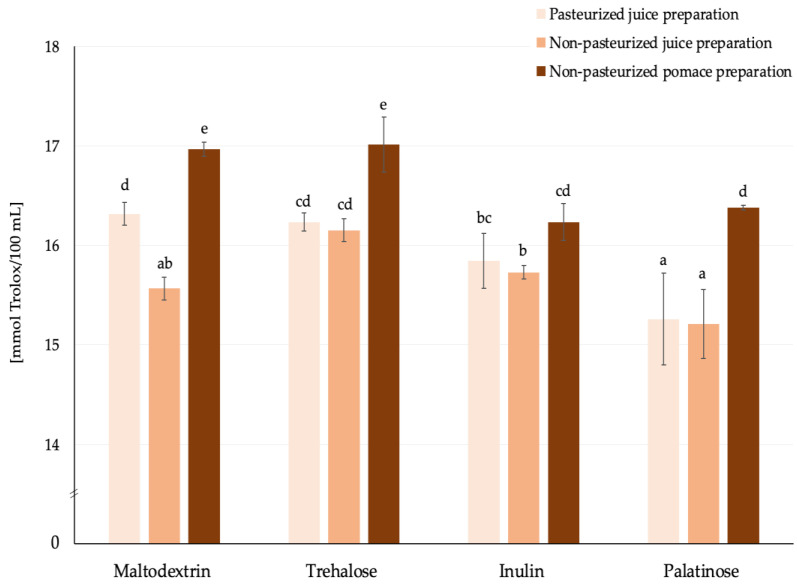
Antioxidant capacity expressed as TEAC ABTS [mmol Trolox/100 mL] of prototype beverages reconstituted from rosehip powders prepared with various carriers by spray drying; ^a,b,c,…^—different small letters represent statistically significant differences between samples (NIR test; *p* ≤ 0.05).

The antioxidant capacity, measured by TEAC ABTS analysis, was evaluated for prototypes of functional beverages formulated from spray-dried rosehip powders selected on the basis of the above-discussed criteria. Phenolics (mainly tannins, flavonoids, phenolic acids) and carotenoids (lycopene, β-carotene) present in the rosehip fruit matrix are the main components contributing to its antioxidant capacity [2]. However, based on our analysis of the powders, no lycopene and β-carotene were present (<0.001 mg/kg). It is also likely that ascorbic acid is another compound, among others, that may contribute to the bioactive properties evaluated in the beverages (*r* = 0.79). Considering the matrix used for powder preparation (regardless of the carrier type applied), it was observed that beverages formulated with non-pasteurized pomace powder exhibited the highest average TEAC ABTS values. This slight difference could be attributed to the highest vitamin C content (Figure 4) in samples from pomace. It should be noted, however, that although the differences appeared to be statistically significant in some cases, the values were relatively comparable, and there was no clear pattern that could substantially distinguish the products (Figure 5).

The screening results indicated that non-pasteurized pomace preparation powders derived from rosehip by-products hold significant potential as valuable ingredients in the formulation of functional beverages, as these drinks exhibit bioactive properties that are comparable to, or even superior to, those observed in beverages produced from juice-derived powders.

#### 2.3.2. Size of Particles 

In the food industry, powders with a higher solubility are particularly sought after, as they allow a more efficient reconstitution in water or other liquids. High solubility is essential to ensure the desired functionality of powders, including instant beverages. The ability to enhance solubility often depends on a detailed understanding of the physical and chemical properties of the powders. This becomes particularly critical when different types of carriers are used to improve product functionality, as the specific attributes of the carriers and their interactions with matrices such as fruit-based components can significantly influence dissolution behavior. Despite their importance, there is limited information in the literature about the effects of interactions between different fruit-based matrices and carriers on the properties of beverages. A particle-size analysis could serve as a valuable tool to assess the size distribution of particles in solutions produced with various carriers and provide insights into their potential impact on beverage properties. The particles in the liquid prototype beverages can be characterized in terms of hydrodynamic diameter (*D_h_*) which also includes the measurement of the immediate surrounding environment [46]. Previously, Calle et al. [46] observed large particles (>170 nm) in fruit juices with an intermediate polydispersity. In the prototype beverages of the present study, the particle size ranged from 34.15 nm to about 7363.55 nm, corresponding to NP-PP with maltodextrin and NP-JP with trehalose, respectively (Table 3).

Additionally, the control samples consisted of the same proportion of single carriers dispersed in water as a solvent. According to particle size, the resolubilized carriers can be classified into two groups: (1) <560 nm—maltodextrin and trehalose; and (2) between 1500 and 2050 nm, i.e., inulin and palatinose. It has been previously shown that the composition of the carriers, including their molecular size, is a significant factor influencing particle size [47,48]. Additionally, the process of spray drying itself, particularly the inlet air temperature, significantly affects the particle size of the products obtained by drying [49,50]. It should be also highlighted that the spray drying parameters used for plant-based powders’ production, also on the industrial scale, may significantly moderate their techno-functional properties [51]. These factors are closely linked to the chemical structure of the carriers, which plays a crucial role in determining their behavior during the spray drying process and their final particle characteristics. Overall, in the beverages containing a specific carrier and a rosehip preparation (P-JP, NP-JP and NP-PP) in powder form, the products were ranked according to particle size as follows: maltodextrin, inulin, palatinose and trehalose (Table 3).

Going into the details, among the matrix types used, the prototype beverages composed of non-pasteurized juice preparation (NP-JP) and maltodextrin, trehalose and palatinose had a greater particle size than those containing pasteurized juice, except inulin. Previous studies have suggested that pasteurization reduces the particle size in apple juice [52]. This has been attributed to the disruptive effect of hydrodynamic stresses leading to the fragmentation of plant tissue, increasing the number of small particles. In the present study, the preparation process using XAD-16 resin likely altered the particle size, and the application of carriers before drying played a key role in the changes to the particle size in powders. It was also noted that small particles in the liquid feed could agglomerate. Interestingly, the particle size trend of products made from non-pasteurized pomace preparations (NP-JP) was generally like those obtained from pasteurized juice preparations. What is more, particle–particle interactions between preparations with a particular composition and carriers may affect the formation of larger-size globules [49]. Thus, the recognition of the effect on particles of different preparation and carrier types should be extended.

Previously, it was speculated that the particle size of the powders may affect their solubility. In the present study, the correlation between solubility values and particle size was *r* = 0.5919, suggesting only a moderate positive relationship. This suggests that larger particle sizes may also contribute to a slightly higher solubility. Further investigation is needed to better understand the mechanisms underlying this relationship and its implications for powder functionality in food and beverage applications.

## 3. Material and Methods

### 3.1. Material

Rosehip (*Rosa canina* L.) fruit (70 kg) was purchased from ‘Szkółka Drzew, Krzewów Owocowych i Róż’ (Kostrzyn Wielkopolski, Poland) and used for juicing with enzymation (50 °C, 16 h) [18]. Half of the juice was pasteurized using a tubular heat exchanger (APV, Delta 4.50.1; flow: 100 L/h; 88 °C; 20 s). After juicing, the extraction of the obtained pomace was conducted at 50 °C for 4 h with 36 L of water, and the slurry was pressed at 180 bar. The obtained juice and pomace were submitted to obtain preparations.

### 3.2. Methods

#### 3.2.1. Obtainment of Preparations

In order to produce the preparations (P) based on pasteurized juice (P-JP) and non-pasteurized juice (NP-JP) and non-pasteurized pomace extract (NP-PP), phenolic compound adsorption and sugar removal were carried out using an XAD-16 resin, preconditioned with methanol and water, following the protocols described by Schieber et al. [53] and Kammerer et al. [54]. The supernatant was applied to the column, and sugars were eluted with water until the pale yellow sugary eluate became colorless. Subsequently, phenolics were desorbed from the XAD-16 resin using ethanol. Ethanol was then evaporated using a Laborota 20-Control rotary evaporator (Heidolph, Schwabach, Germany) at 40 °C, concentrating the aqueous extract to a final volume of 2 L. The resulting preparations were divided into 500 mL aliquots and subjected to drying processes.

#### 3.2.2. Drying

Approximately 500 mL of pasteurized juice preparation (P-JP; 8.2 °Bx), non-pasteurized juice preparation (NP-JP; 8.4 °Bx) and non-pasteurized pomace preparation (NP-PP; 8.2 °Bx) was mixed with 20% (*w*/*w*) of maltodextrin (Pepees S.A., Łomża, Poland) and unconventional carbohydrate carriers: trehalose (Hayashibara Co., Okayama, Japan), palatinose (PST-N, isomaltulose; Beneo-Palatinit GmbH, Mannheim, Germany) and inulin (Beneo-Orafti, Oreye, Belgium). The mix of each preparation and carrier was submitted to freeze-drying (FreeZone, Labconco Corp., Kansas City, MO, USA; 24 h, under a reduced pressure of 65 Pa, temperature of the chamber and heating plate: −60 °C/+24 °C) and spray drying (Mini Spray dryer B-290 Advanced, Buchi, Flawil, Switzerland; two-fluid nozzle atomizer with inside diameter of 1.5 mm; spray drying parameters were adjusted to an inlet air temperature of 130 °C; Table 4). The addition of palatinose to the rosehip preparation required lower aspirator values due to the stickiness issue in the drying chamber. The control samples were considered as a freeze-dried preparation without the addition of carriers, as the composition of the samples made it impossible to obtain powders without carriers using spray drying. The powders were made in two technological repetitions (*n* = 2).

#### 3.2.3. Preparation of Beverage Prototypes

Each spray-dried product obtained from rosehip juice and pomace preparations was dissolved in cold tap water (ambient temperature) using the proportion of 20 mg of powder to 1 mL of water. The prototype solution was stirred for 90 s. The prototype beverages were prepared in duplicate (*n* = 2) and directly submitted to the analyses.

#### 3.2.4. Basic Chemical Composition of Raw Material

The dry matter (DM) was examined by the vacuum-oven method according to Figiel [55] at 80 °C for 24 h and was calculated as a difference between 100% and moisture content. The titratable acidity (TA) was examined using a TitroLine 5000 (Xylem Analytics GmbH, Weilheim, Germany) with 0.1 mol/L NaOH to pH 8.1, and the results were presented as mg malic acid/100 g of sample (PN-EN 12145:2000) [56]. Ash and ascorbic acid content were determined according to norm PN-90/A-75101/08 and PN-A-04019 [56], and pectin content (g/100 g) was measured using the Morris method [56]. Total phenolic compound (TPC) content was examined with the use of Folin–Ciocalteu reagent [57]. For the carotenoids analysis, the freeze-dried preparations (1 g) were extracted in an ultrasonic ice bath for 10 min using 1 mL hexane (50:50, *v*/*v*) with 0.1% BHT with the detailed procedure previously described [18]. After centrifugation (4400 rpm, 10 min, 4 °C), the supernatant was filtered and concentrated under nitrogen to 40 µL, then stored at 4 °C until HPLC analysis. Chromatography was performed on an Agilent 1220 LC system with a Luna C_18_ column (250 × 4.6 mm, 5 µm), using acetone (A) and methanol (B) as mobile phases. A gradient of 5–55% of A over 15 min was applied with a 1 mL/min flow rate. Lycopene and β-carotene were quantified at 450 nm with external calibration (LOQ: 100 µg/kg and 150 µg/kg, respectively). All measurements were made in duplicate (*n* = 2).

#### 3.2.5. Physico-Chemical Properties of Powders

The water activity (*a_w_*) was measured using the Dew Point water activity meter (Water Activity Meter, 4TE, AQUA LAB, Pullman, WA, USA) at 25 °C in duplicate (*n* = 2).

#### 3.2.6. Color

The color of the powders was measured in triplicate (*n* = 3) (Minolta Chroma Meter CR-400 colorimeter; Minolta Co. Ltd., Osaka, Japan) following the CIE L*a*b* system. The browning index (BI) of the powders was calculated using the equation from Palou et al. [58], while the yellowness index (YI) was determined based on the method described by Rhim et al. [59].

#### 3.2.7. Water Absorption Capacity (WAC) and Water Solubility Index (WSI)

The WAC and WSI measurements were performed according to Vicente et al. [60] with slight modifications. For the WAC analysis, a 0.5 g sample of dry powder (DM) was combined with 5 mL of water in centrifuge tubes, vortexed for 60 sec, allowed to stand at room temperature for 10 min, then vortexed again (60 s) before being centrifuged for 25 min at a speed of 3000× *g*. The supernatant was carefully discarded, and the remaining material was weighed. The results were calculated as the amount of water held in g/g of powder DM. For the WSI analysis, a similarly prepared mixture was heated at 95 °C for 15 min, then cooled to room temperature and finally centrifuged (25 min; 3000× *g*). The supernatant was poured onto a previously weighed Petri dish, weighed, placed in a dryer at 110 °C overnight and weighed again after the water had been removed. The WSI was expressed as g of soluble solids per 100 g of powder DM.

#### 3.2.8. Antioxidant Capacity In Vitro

The antioxidant capacity of rosehip powders and beverages was examined by TEAC ABTS [61] assay using a Synergy H1 spectrophotometer (BioTek Instruments Inc., Winooski, Vermont, USA). The determination was made in duplicate (*n* = 2), and the results were expressed as mmol Trolox Equivalent (TE) per 100 g of DM for powders or 100 mL of beverage.

#### 3.2.9. Particle-Size Analysis

The average size of particles in beverages from rosehip powders was determined using a dynamic light scattering (DLS) technique using the nanoPartica SZ-100V2 Series (Horiba, Kyoto, Japan). The measurements were performed at a detector angle of 90°. The temperature during measurements was 25 °C. The result was presented as an average value for three measurements (*n* = 3) with standard deviation (SD).

### 3.3. Statistical Analysis

The statistical analysis of the average values was performed using the STATISTICA 13 program (StatSoft, Tulsa, OK, USA) by the analysis of variance (ANOVA) and the NIR test (*p* ≤ 0.05).

## 4. Conclusions

In summary, this study demonstrated the feasibility of producing powdered juice and pomace preparations from rosehip with a moisture content below 10% and water activity below 0.4, ensuring microbiological stability. The techno-functional properties were primarily influenced by the carrier type, while color attributes, such as the yellowness and browning indexes, depended more on the matrix composition, particularly the ascorbic acid content. Spray drying turned out to be a competitive alternative to freeze-drying, yielding powders with a lower moisture content, reduced water activity and higher solubility, especially when palatinose was used as a carrier, enhancing the functional properties of the powders for beverage applications. Water absorption capacity can be modified by the drying technique, notably for inulin as a carrier. Interestingly, non-pasteurized pomace powders had a higher antioxidant capacity than their juice counterparts. The antioxidant capacity of juices and powders revealed a similar trend, highlighting pomace as a promising matrix for beverage production. Particle-size analysis suggested potential interactions between carriers and matrix components, as reflected in the distinct trends observed in carrier-only samples. However, the mechanisms underlying these differences remain unclear. Further investigation is needed to elucidate these interactions and evaluate their impact on the performance of the powders in food and beverage applications. This study has highlighted the potential for new applications of rosehip pomace, providing a foundation for future product development, as the use of rosehip pomace has been shown to yield powders with chemical properties comparable to, or even superior to, those derived from juice. For the scalability of the lab-scale process, further investigations are required to elucidate these interactions and evaluate their influence on the performance of the powders in food and beverage applications. Additionally, consumer acceptability should be assessed for the final, upscaled product.

## Figures and Tables

**Table 1:** The basic chemical composition of preparations obtained from rosehip juice and pomace.

	Pasteurized Juice Preparation	Non-Pasteurized Juice Preparation	Non-Pasteurized Pomace Preparation
Dry matter [%]	6.66 ± 0.01 ^b^	6.06 ± 0.01 ^a^	6.11 ± 0.04 ^c^
Acidity [mg malic acid/100 g]	0.56 ± 0.01 ^a^	0.58 ± 0.04 ^a^	0.63 ± 0.01 ^b^
Pectins [%]	ND	ND	ND
Ascorbic acid [mg/100 g]	7.74 ± 0.01 ^a^	17.06 ± 0.54 ^c^	15.49 ± 0.53 ^b^
Total phenolic compounds [g GAE/L]	9.09 ± 0.41 ^c^	8.13 ± 0.19 ^c^	6.92 ± 0.25 ^a^
β-Carotene [mg/kg]	<0.001	<0.001	2.20 ± 0.17
Lycopene [mg/kg]	<0.001	<0.001	0.40 ± 0.05

ND—not detected; GAE—gallic acid equivalent; ^a,b,c^—different letters in the rows indicate statistically significant differences (NIR test; *p* ≤ 0.05).

**Table 2:** Moisture content (*Mc*), water activity (*a_w_*), color (CIE L*a*b*), browning index (BI) and yellowness index (YI) of rosehip powders gained after spray drying and freeze-drying.

Matrix	Drying Technique	Carrier Type	*Mc*	*a_w_*	Color			BI	YI
			[%]	[–]	L*	a*	b*	[%]	[%]
Pasteurized juice preparation	FD	—	4.22 ± 0.54 ^A^	0.143 ± 0.005 ^A^	65.58 ± 0.16 ^A^	13.84 ± 0.06 ^B^	32.28 ± 0.08 ^A^	81.42 ± 0.56 ^B^	70.32 ± 0.36 ^B^
Non-pasteurized juice preparation			8.87 ± 0.10 ^C^	0.236 ± 0.001 ^B^	75.26 ± 0.33 ^C^	10.84 ± 0.03 ^A^	32.24 ± 0.18 ^A^	65.29 ± 0.06 ^A^	61.19 ± 0.07 ^A^
Non-pasteurized pomace preparation			6.55 ± 0.01 ^B^	0.285 ± 0.004 ^C^	70.26 ± 0.01 ^B^	14.87 ± 0.02 ^C^	38.87 ± 0.01 ^B^	93.01 ± 0.06 ^C^	79.03 ± 0.03 ^C^
Pasteurized juice preparation	SD	Maltodextrin	4.26 ± 0.16 ^j^	0.058 ± 0.003 ^c^	76.78 ± 1.04 ^d-g^	4.54 ± 0.44 ^ef^	19.63 ± 1.02 ^c^	33.32 ± 1.67 ^c^	36.51 ± 1.41 ^c^
		Trehalose	2.72 ± 0.53 ^f–h^	0.082 ± 0.003 ^e^	81.68 ± 2.58 ^g-j^	4.19 ± 0.02 ^de^	20.14 ± 0.35 ^cd^	31.57 ± 0.54 ^bc^	35.24 ± 0.48 ^c^
		Inulin	3.39 ± 0.15 ^i^	0.069 ± 0.001 ^d^	76.17 ± 2.42 ^d–g^	5.69 ± 0.10 ^g–i^	22.33 ± 0.47 ^e^	39.59 ± 0.50 ^d^	41.90 ± 0.44 ^de^
		Palatinose	2.11 ± 0.21 ^c–e^	0.142 ± 0.004 ^j^	75.80 ± 4.48 ^d–g^	5.26 ± 0.73 ^gh^	22.48 ± 2.37 ^e^	39.59 ± 2.32 ^d^	42.32 ± 1.96 ^de^
	FD	Maltodextrin	2.88 ± 0.47 ^g–i^	0.060 ± 0.000 ^c^	67.83 ± 4.66 ^b^	9.89 ± 0.32 ^m^	30.87 ± 1.36 ^ij^	70.02 ± 2.32 ^g^	65.06 ± 1.59 ^h^
		Trehalose	2.95 ± 0.09 ^g–i^	0.096 ± 0.003 ^fg^	73.92 ± 0.30 ^b–f^	8.31 ± 0.07 ^jk^	28.77 ± 0.19 ^h^	56.59 ± 0.78 ^f^	55.61 ± 0.61 ^g^
		Inulin	1.46 ± 0.25 ^b^	0.041 ± 0.001 ^ab^	77.77 ± 3.97 ^d–h^	5.88 ± 0.00 ^hi^	22.68 ± 0.70 ^e^	39.46 ± 1.11 ^d^	41.69 ± 0.85 ^de^
		Palatinose	6.06 ± 0.01 ^l^	0.322 ± 0.001 ^n^	55.88 ± 3.02 ^a^	10.18 ± 0.28 ^m^	29.13 ± 0.03 ^hi^	85.44 ± 7.21 ^h^	74.89 ± 4.41 ^i^
Non-pasteurized juice preparation	SD	Maltodextrin	2.57 ± 0.11 ^e–g^	0.099 ± 0.007 ^gh^	76.86 ± 6.62 ^d–g^	3.13 ± 0.02 ^ab^	15.92 ± 0.76 ^b^	25.80 ± 1.24 ^a^	29.63 ± 1.14 ^ab^
		Trehalose	0.78 ± 0.09 ^a^	0.103 ± 0.005 ^h^	85.10 ± 2.46 ^ij^	3.44 ± 0.28 ^bc^	15.89 ± 1.03 ^b^	23.22 ± 0.94 ^a^	26.66 ± 0.94 ^a^
		Inulin	1.43 ± 0.28 ^b^	0.097 ± 0.008 ^gh^	68.20 ± 7.23 ^b^	2.66 ± 0.02 ^a^	13.71 ± 0.85 ^a^	24.97 ± 1.36 ^a^	28.81 ± 1.25 ^ab^
		Palatinose	1.80 ± 0.21 ^bc^	0.163 ± 0.005 ^k^	77.88 ± 0.88 ^d–h^	5.81 ± 0.29 ^g–i^	19.29 ± 0.35 ^c^	33.49 ± 1.26 ^c^	35.41 ± 1.01 ^c^
	FD	Maltodextrin	2.16 ± 0.34 ^c–e^	0.035 ± 0.001 ^a^	81.31 ± 0.94 ^g–j^	5.19 ± 0.08 ^fg^	25.07 ± 0.02 ^f^	40.86 ± 0.68 ^d^	44.06 ± 0.56 ^e^
		Trehalose	4.36 ± 0.27 ^j^	0.164 ± 0.004 ^k^	79.38 ± 0.05 ^f–i^	7.87 ± 0.05 ^j^	28.04 ± 0.19 ^gh^	50.05 ± 0.43 ^e^	50.46 ± 0.36 ^f^
		Inulin	0.82 ± 0.03 ^a^	0.035 ± 0.000 ^a^	84.19 ± 0.18 ^h–j^	3.61 ± 0.06 ^b–d^	18.47 ± 0.04 ^c^	27.44 ± 0.08 ^ab^	31.34 ± 0.02 ^b^
		Palatinose	4.92 ± 0.22 ^k^	0.211 ± 0.003 ^l^	72.59 ± 2.23 ^b-e^	9.55 ± 0.11 ^lm^	32.50 ± 0.57 ^j^	67.62 ± 1.21 ^g^	63.98 ± 0.85 ^h^
Non-pasteurized pomace preparation	SD	Maltodextrin	3.14 ± 0.27 ^hi^	0.095 ± 0.002 ^fg^	78.58 ± 2.39 ^e–i^	4.09 ± 0.03 ^c–e^	19.64 ± 0.40 ^c^	32.05 ± 0.43 ^c^	35.71 ± 0.37 ^c^
		Trehalose	2.27 ± 0.09 ^c–f^	0.119 ± 0.003 ^i^	75.38 ± 6.06 ^c–g^	5.74 ± 0.59 ^g–i^	21.67 ± 2.15 ^de^	38.85 ± 0.90 ^d^	41.03 ± 0.79 ^de^
		Inulin	2.35 ± 0.04 ^d–f^	0.089 ± 0.001 ^ef^	75.84 ± 3.87 ^d–g^	5.68 ± 0.06 ^g–i^	23.12 ± 0.74 ^e^	41.24 ± 1.04 ^d^	43.58 ± 0.83 ^de^
		Palatinose	2.02 ± 0.35 ^cd^	0.144 ± 0.003 ^j^	76.96 ± 1.87 ^d–g^	5.25 ± 0.81 ^gh^	21.96 ± 0.83 ^de^	38.12 ± 3.44 ^d^	40.83 ± 2.59 ^d^
	FD	Maltodextrin	1.94 ± 0.06 ^b–d^	0.045 ± 0.002 ^b^	71.29 ± 2.36 ^b–d^	6.16 ± 0.07 ^i^	27.86 ± 0.39 ^gh^	54.89 ± 1.52 ^f^	55.86 ± 1.11 ^g^
		Trehalose	3.19 ± 0.23 ^hi^	0.086 ± 0.000 ^e^	68.67 ± 1.61 ^bc^	8.05 ± 0.00 ^j^	26.67 ± 0.15 ^fg^	56.84 ± 1.33 ^f^	55.50 ± 1.01 ^g^
		Inulin	2.61 ± 0.17 ^e–g^	0.256 ± 0.003 ^m^	86.62 ± 1.10 ^j^	4.48 ± 0.65 ^e^	22.52 ± 1.31 ^e^	33.38 ± 3.06 ^c^	37.16 ± 2.63 ^c^
		Palatinose	3.91 ± 0.39 ^j^	0.069 ± 0.003 ^d^	78.71 ± 0.08 ^e–i^	8.98 ± 0.04 ^kl^	30.93 ± 0.03 ^ij^	57.31 ± 0.17 ^f^	56.15 ± 0.11 ^g^

SD—spray drying, FD—freeze-drying; ^A,B,C,…^—different capital letters within groups (controls) indicate statistically significant difference (NIR test; *p* ≤ 0.05); ^a,b,c,…^—different small letters within groups (pasteurized juice preparation, non-pasteurized juice preparation, non-pasteurized pomace preparation) indicate statistically significant difference (NIR test; *p* ≤ 0.05).

**Table 3:** Particle size [nm] of beverages prepared from rosehip spray-dried powders with various carriers.

Carrier Type	Control *	Pasteurized Juice Preparation	Non-Pasteurized Juice Preparation	Non-Pasteurized Pomace Preparation
Maltodextrin	102.6 ± 11.17 ^a^	164.55 ± 3.46 ^a^	4278.85 ± 435.22 ^e^	34.15 ± 2.90 ^a^
Trehalose	555.6 ± 77.22 ^a^	3252.55 ± 192.97 ^d^	7363.55 ± 1393.50 ^g^	5822.90 ± 340.54 ^f^
Inulin	1525.05 ± 144.18 ^b^	2932.95 ± 41.93 ^cd^	2739.55 ± 130.89 ^cd^	3519.80 ± 289.21 ^de^
Palatinose	2026.35 ± 235.96 ^bc^	3568.70 ± 57.28 ^de^	5459.70 ± 757.88 ^f^	3505.55 ± 79.13 ^de^

*—control—no rosehip preparation added; ^a,b,c,…^—different small letters represent statistically significant differences between samples (NIR test; *p* ≤ 0.05).

**Table 4:** Spray drying parameters for production of rosehip preparation powders depending of carrier type used.

Sample	Carrier	Inlet Air Temperature [°C]	Outlet Air Temperature [°C]	Volume Flow * [m^3^/h]	Atomization Air Flow [m^3^/h]	Feed Flow Rate [mL/min]
P-JP	maltodextrin	130	84 ± 1	35	35	3.5
	trehalose		83 ± 3	35		3.5
	inulin		84 ± 3	35		3.5
	palatinose		77 ± 2	27		3.5
NP-JP NP-PP	maltodextrin		86 ± 4	35		4.0
	trehalose		84 ± 2	35		3.5
	inulin		83 ± 2	35		3.5
	palatinose		74 ± 2	27		3.5

* Aspirator, gas flow rate; P-JP—pasteurized juice preparation, NP-JP—non-pasteurized juice preparation; NP-PP—non-pasteurized pomace preparation.

## Data Availability

Data are contained within the article.
